# Poly(Hydroxyethyl Methacrylate) Immunoaffinity Cryogel Column for the Purification of Human Immunoglobulin M

**DOI:** 10.3390/gels6010004

**Published:** 2020-01-29

**Authors:** Monireh Bakhshpour, Aykut Arif Topcu, Nilay Bereli, Huseyin Alkan, Adil Denizli

**Affiliations:** 1 Department of Chemistry, Biochemistry Division, Hacettepe University, 06800 Ankara, Turkey; b.monir@hacettepe.edu.tr (M.B.); bereli@hacettepe.edu.tr (N.B.); 2 Department of Chemistry, Aksaray University, 68100 Aksaray, Turkey; aykuttopcu81@gmail.com; 3 Department of Chemistry, Dicle University, 21280 Diyarbakır, Turkey; mhalkan@dicle.edu.tr

**Keywords:** hIgM purification, adsorption, immunoaffinity, cryogel column

## Abstract

Human immunoglobulin M (hIgM) antibodies are considered as hopeful tools for diseases therapy. Therefore, chromatography approaches are used to purify hIgM with a single step. In this study, we prepared a poly(hydroxyethyl methacrylate) based immunoaffinity p(HEMA-I) cryogel column by using cyanamide to immobilize antihuman immunoglobulin on the p(HEMA) cryogel for purification of hIgM in aqueous solution and artificial human plasma. The characterization of the p(HEMA) cryogel column was performed by using a scanning electron microscope (SEM), micro-computerized tomography (µ-CT), Fourier transform infrared spectroscopy (FTIR), swelling degree and macro-porosity. Further, the optimizations of various parameters were performed such as, pH, ionic strength, temperature and concentration of hIgM in aqueous solutions. In addition, the Langmuir adsorption model was supported by experimental results. Maximum adsorbed amount of hIgM corresponded to 11.1 mg/g at pH 5.75 [morpholino ethanesulfonic acid (MES buffer)]. Our results indicated that the p(HEMA-I) cryogel column can be reused at least 10 times without significant loss in adsorption capacity. As a natural source, artificial human plasma was selected for hIgM adsorption and the purity of hIgM was evaluated using sodium dodecyl sulfate polyacrylamide gel electrophoresis (SDS-PAGE).

## 1. Introduction 

In the past, immunoglobulins (Igs) were used as a therapeutic agent to treat the diphtheria disease and this approach leads to the use of an antibody-based therapy [1,2]. Today, development of hybridoma technology dramatically increased the use of Igs in different biopharmaceutical areas for medical purposes [1,3,4]. Different ligands such as 2-mercapto-l-methylimidazole and 2-mercapto-benzimidazole have been previously utilized to develop Igs that, subsequently, showed a high separation efficiency during immunoglobulin G (IgG) purification [5,6]. Besides, immunoglobulin Y (IgY) was purified by resins from chicken with high recovery and purity [7,8]. Immunoglobulin M (IgM), the first produced antibody by the immune system to combat or stop an infection, is used in the stem cell research field as well as for treatment and management of life-threatening diseases [9,10,11,12,13]. hIgM dominantly exists in human serum (0.4 mg/mL) in the form of a cyclic pentamer with 950 kDa molecular weight [14]. Therapeutic studies require high purity and affordable downstream processing; therefore, method selection of hIgM purification plays a pivotal role in medical fields [4,15].

Precipitation and chromatographic methods are extensively used in hIgM purification from biological fluids. Polyethylene glycol (PEG) plays a key role in a simple enrichment process, for isolation of large molecules like hIgM even in large scale purification [1,4,16,17]. However, precipitation is time-consuming, ensures poor selectivity and the purification yields of hIgM are not highly pure for therapeutic studies [1,4]. To overcome these limitations of precipitation, chromatographic approaches such as hydroxyapatite, anion-exchange, hydrophobic and affinity chromatography [6,18,19,20,21,22,23,24] can be used. Affinity chromatography is usually preferred due to its high selectivity, which allows us to purify antibodies with a single step [4].

Immunoaffinity chromatography is a specific type of affinity chromatography, which is based on the certain and firm antigen–antibody interactions. Before the purification process, antigens or antibodies are covalently immobilized on support materials such as agarose, cellulose, acrylamide or chromatographic resins [25,26,27,28,29,30,31]. However, due to the low binding constant and large molecular size of hIgM, macroporous support materials like monoliths are preferably used during hIgM purification [17,21,22,25]. Cryogels have a great potential as an affinity separation tool for the isolation of large molecules such as deoxyribonucleic acid (DNA), antibodies, proteins and cells [32,33,34,35,36]. 

Herein, we developed an immunoaffinity cryogel column for single step separation of a human (hIgM) with high purity. For this purpose, a p(HEMA) based cryogel column was prepared via free-radical polymerization under semi-frozen conditions. After that, a p(HEMA) cryogel column was activated by cyanamide and then, anti-hIgM was attached covalently to the p(HEMA) cryogel column. Characterization of anti-hIgM attached the p(HEMA-I) column and p(HEMA) cryogel column were investigated by SEM, micro-computerized tomography (µ-CT), FTIR, a swelling test and macroporosity. In addition, the effect of organic and inorganic buffer solutions (pH 4.0–8.0), initial hIgM concentration (0–0.5 mg hIgM/mL), temperature effect (4–45 °C) and ionic-strength [(0–0.5 mM, sodium chloride (NaCI) solution] on hIgM binding capacity of the p(HEMA-I) cryogel column were investigated. Moreover, p(HEMA-I) cryogel column-capacity to purify hIgM from artificial human plasma was also determined. Further, the purity of hIgM was investigated by SDS-PAGE.

## 2. Results and Discussion 

### 2.1. Characterization

The FTIR spectrum of the p(HEMA) cryogel column was given in the Supplementary Materials and our results were in accordance with previous studies [37,38]. 

**Figure 1:**
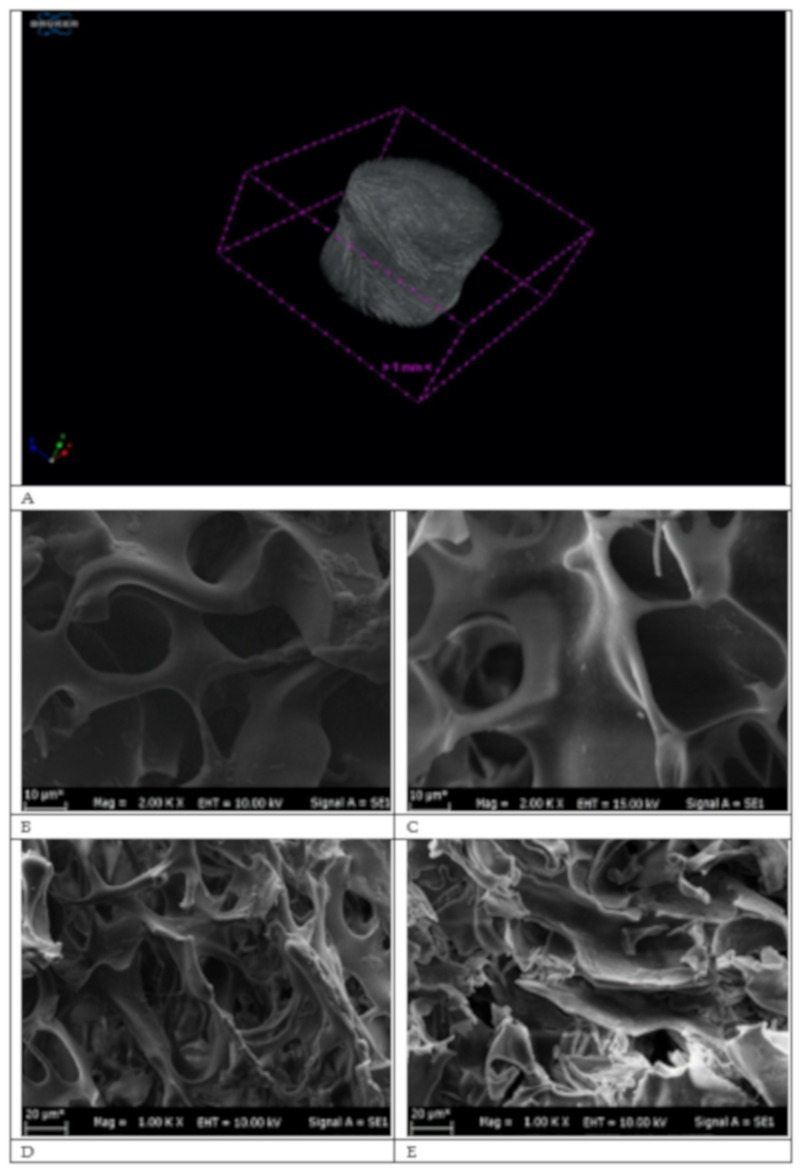
μCT measurement of the p(HEMA) cryogel column (**A**). SEM images of cryogel columns: (**B**,**D**) the p(HEMA-I) and (**C**,**E**) the p(HEMA) cryogel columns.

µ-CT is a technique that analyzes the distribution of pores and the structure of the cryogel column with a high-resolution and a non-destructive 3-D format. X-rays were sent to the cryogel with an angle of 360° at intervals of 0.4. As seen in Figure 1A, the µ-CT image of the p(HEMA) cryogel was given and according to the µ-CT analysis (CT Analyzer, version: 1.18.4.0), total open porosity (percent) of the p(HEMA-I) column was found 95.2% by volume. Further, cross-section, surface topology and inner sides of p(HEMA) and p(HEMA-I) cryogel columns were investigated with SEM. 

As seen in Figure 1B–E, diameters of the channel macroporous of the p(HEMA) and the p(HEMA-I) cryogels were up to 20 µM, which are much larger than the hydrodynamic radius of hIgM molecules and as illustrated in Figure 1C–E, the p(HEMA-I) immunoaffinity column protected its sponge like morphology, elasticity and pore wall thickness after the immobilization of anti-hIgM. The p(HEMA-I) cryogel can enhance the mass transfer during the adsorption process owing to its macroporous structure [19].

**Figure 2:**
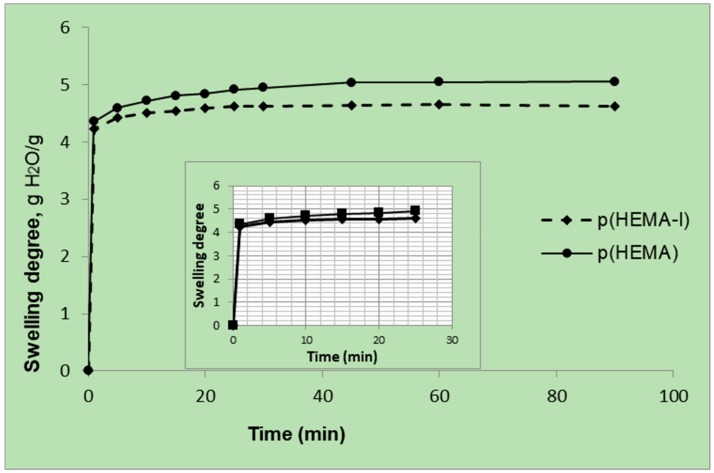
Swelling degree of the p(HEMA) and the p(HEMA-I) cryogel columns.

The equilibrium swelling experiments of cryogel columns were performed at room temperature and repeated three times. The relationship of the swelling degree and time is shown in Figure 2. The cryogels can be easily squeezed by hand to remove the water inside of the macropores. When the squeezed cryogel was put into the water, it swelled in water and maintained its initial shape and size in a few seconds due to its interconnected macroporous structure. The majority of the uptake of water by the p(HEMA) and the p(HEMA-I) cryogel columns occurred within the first 1 min and then reached equilibrium after 15 min. The fast-swelling behavior in the first 1 min might result from the relatively large pore structure of these two cryogel columns, which accelerated the water molecules to diffuse into the pores of cryogels [35,39,40]. Table 1 shows the swelling degree (gH_2_O/g cryogel) and macroporosity (volume %) of the p(HEMA) and the p(HEMA-I) cryogel columns. Swelling degree was decreased during the surface modification, which was carried on the OH groups of the p(HEMA) cryogel in order to immobilize the anti-hIgM molecules. 

### 2.2. Effect of Different Concentrations of Anti-hIgM on Higm Binding

**Figure 3:**
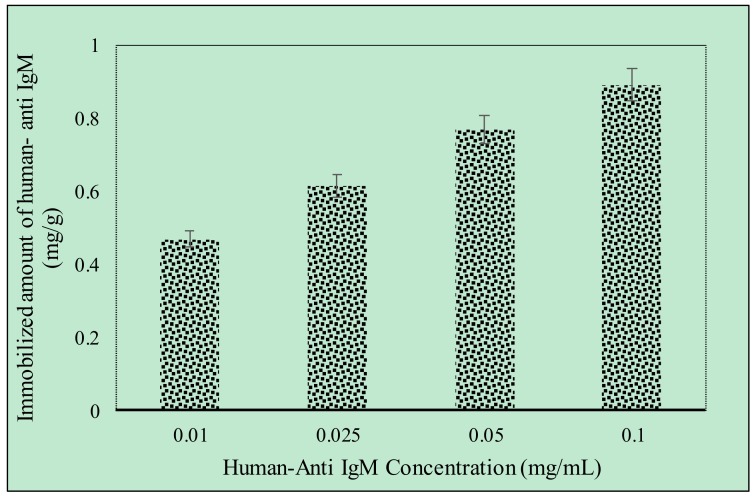
Human-anti IgM immobilization (mg/mL) flow rate of 1 mL/min at room temperature, time; 120 min, *n* = 3.

Different amounts of anti-hIgM (0.01–0.1 mg/mL) were immobilized onto the p(HEMA) cryogel column. As shown in Figure 3, anti-hIgM concentration positively increased the amount of bound anti-hIgM. The maximum anti-hIgM bound amount (0.89 mg/g) was achieved when anti-hIgM concentration was 0.1 mg/mL. The capacity of the p(HEMA) cryogel did not increase with increasing the concentration of anti-hIgM to over 0.1 mg/mL. Therefore, a 0.1 mg/mL anti-hIgM immobilizing the p(HEMA-I) cryogel column was selected for further adsorption studies of hIgM in aqueous solution and artificial human plasma.

### 2.3. Effect of Adsorption Parameters on hIgM Binding

In this part, the effect of pH, hIgM concentration, ionic strength and temperature parameters were investigated for hIgM adsorption. 

**Figure 4:**
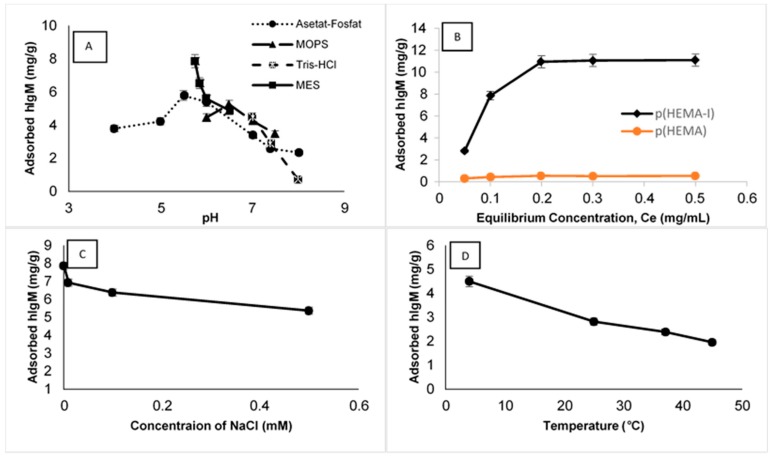
(**A**) pH buffer system for the p(HEMA-I) cryogel column (hIgM concentration 0.1 mg/mL at room temperature), (**B**) effect of hIgM concentration for the p(HEMA-I) and the p(HEMA) cryogel columns (pH 5.75 MES buffer at room temperature), (**C**) ionic strength for the p(HEMA-I) cryogel column (pH 5.75 MES buffer, hIgM concentration 0.1 mg/mL at room temperature) and (**D**) temperature for the p(HEMA-I) cryogel column, pH 5.75 MES buffer, hIgM concentration 0.05 mg/mL, flow rate; 1 mL/min, time; 120 min, anti-hIgM immobilized amount = 0.89 mg/g, *n* = 3.

The effect of pH on hIgM adsorption capacities was examined in the range of pH 4.0–8.0 values. The hIgM adsorption amounts of cryogel columns at different pH values are shown in Figure 4A. At this range of pH, the maximum adsorption capacity was observed at pH 5.75 for the MES buffer and decreased considerably at more alkaline and acidic pH values. This result could be attributed to the specific binding sites of antigen and antibody molecules that are composed of small portions of few amino acids and pH adjustment directly affects the charged groups of amino acid residues. So, over the pH 6.0 values, hIgM adsorption started to decrease owing to repulsions of the same charged groups of hIgM and anti-hIgM molecules.

Different amounts of initial hIgM concentrations (0.05–0.5 mg/mL, pH 5.75 MES buffer at room temperature) were selected to investigate the concentration effect on hIgM adsorption and results are shown in Figure 4B. Adsorbed amounts of hIgM drastically increased with increasing of hIgM concentration and then started to saturate at 0.2 mg/mL hIgM concentration. Maximum hIgM adsorption capacity was observed in an 11.10 mg/g polymer and no significant increment was observed after this value.

The effect of ionic strength on hIgM adsorption by the p(HEMA-I) cryogel column was characterized with hIgM solution prepared in a pH 5.75 MES buffer containing NaCl at different concentrations (0–0.5 mM; Figure 4C). An increase in the hIgM adsorption amount onto the p(HEMA-I) cryogel column was detected upon decreasing NaCl concentration. The minimum adsorbed amount of hIgM (5.35 mg/g) was detected in the presence of a 0.5 mM NaCl solution. Two reasons could explain these results. Firstly, electrostatic interactions are weak interactions and increasing salt concentrations can easily break the electrostatic interactions and salt bridges between the hIgM and anti-hIgM. Secondly, hIgM can lose its solubility in the salt medium owing to its large molecular weight and its tendency to precipitate.

The effect of temperature on hIgM adsorption capacity was investigated between 4 and 45 °C. As the temperature was increased, adsorption capacity of hIgM decreased from 4.49 to 1.95 mg/g (Figure 4D). According to the experimental results, hIgM adsorption was exothermic and hydrophobic interactions were not dominant for the hIgM adsorption.

Experimental results were applied to determine the Langmuir, the Freundlich and the Langmuir–Freundlich adsorption isotherm models. As shown in Table 2, a high correlation coefficient (R^2^  =  0.99) in the Langmuir isotherm indicates that the system suited the Langmuir isotherm model. For this reason, hIgM binding on the p(HEMA-I) cryogel column is monolayer binding. The Langmuir model describes the homogeneous adsorption, which occurs in the monolayer while the Freundlich isotherm model is based on multilayer adsorption and refers to heterogeneous adsorption.

### 2.4. hIgM Purification from Artificial Human Plasma

**Figure 5:**
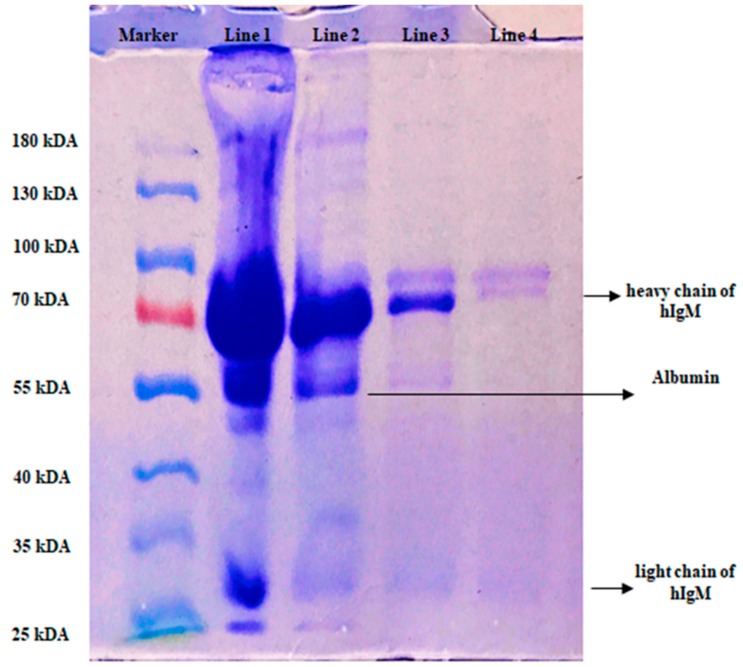
SDS-PAGE analysis of hIgM Line 1; 1/2 eluted of the artificial human plasma, Line 2; final concentration of plasma, Line 3; eluted hIgM and Line 4; hIgM solution (0.1 mg/mL).

In this study, we used artificial human plasma as a natural source of hIgM and the image of gel was shown in Figure 5. Line 1; the eluted of the initial concentration of hIgM, Line 2; final concentration, Line 3; desorption and Line 4; 0.1 mg/mL hIgM concentration and as seen in the Figure 5, hIgM was highly purified by the p(HEMA-I) column.

### 2.5. Literature Comparison with the Present Study 

Brne et al. [41] prepared ion-exchange methacrylate based short monolithic columns for the purification of IgG and IgM and maximum adsorbed amount of IgM was found over 20 mg/mL. In another study, a hexamer peptide ligand was used for the purification of IgM by using affinity chromatography and maximum adsorption capacity was calculated in the range of 4.6–13.1 mg/mL owing to a large molecular structure of IgM [23]. Another study was based on using mixed mode chromatography with different ligands including ABI-4FF [14]. According to experimental results, separation of IgM from a hybridoma cell supernatant was highly achieved with the ABI-4FF ligand with good recovery and purity. Hennicke et al. [42] investigated pH stability to optimize the elution conditions for affinity purification of monoclonal IgM, resulting in a fast and efficient single-step downstream strategy. They optimized single-step purification strategy for high purity, high yield and retained antigen-binding capacity. They combined pH 3.5 with a high salt concentration to prevent aggregation during elution. In the other study, they reported the impact of temperature and pH on recombinant human IgM quality attributes and productivity [43]. They showed the influence of temperature and pH on cell-specific productivity and IgM quality attributes. They performed biphasic temperature and pH shift experiments as batch cultures in DASGIP® bioreactors. They used an internally developed recombinant IgM producing chinese hamster ovary (CHO) cell line. They evaluated all quality attributes by densitometric and chromatographic methods. They reported that the reduction of cultivation temperature severely reduced IgM titers, while pH variation had no impact. In contrast, IgM quality was not significantly influenced by bioprocessing parameters. In the present study, we prepared p(HEMA) based cryogel column under semi-frozen conditions and anti-hIgM was immobilized on the p(HEMA) cryogel, which was used as an immunoaffinity column for hIgM purification in aqueous solution and artificial human plasma with a single step. 

### 2.6. Reusability of the p(HEMA-I) Column

**Figure 6:**
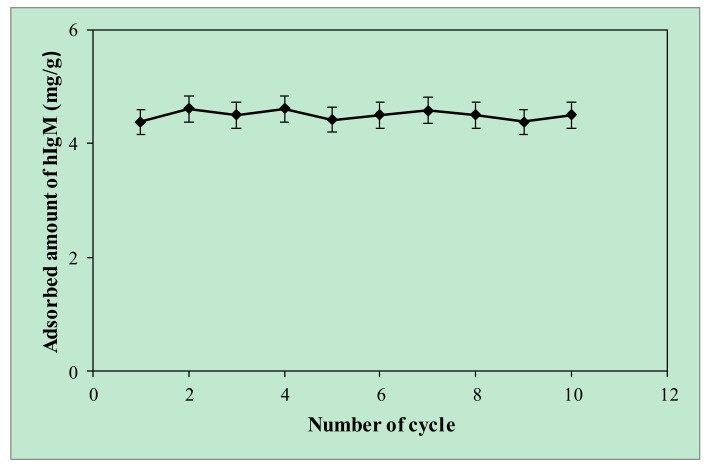
Reusability studies (pH; 5.75 MES buffer, hIgM concentration; 0.1 mg/mL, flow rate; 1 mL/min, time; 120 min, at room temperature).

Today, cost-effective protein purification methods are in high demand for protein science and low-cost adsorbents attract the attention of researchers, who attempt to use and develop cost-effective adsorbents in an attempt to decrease the cost of adsorption and achieve better adsorption performance [44]. Thereby, the reusability of an affinity adsorbent is a key issue to investigate the adsorption performance of an adsorbent during the whole adsorption process including adsorption–desorption–regeneration cycles and plays a pivotal role in determining of economic feasibility analysis. In addition, high regeneration efficiency of an adsorbent is a generally important feature, which makes it for more suitably used in adsorption studies. For these reasons, the same p(HEMA-I) cryogel column was repeatedly used in 10 adsorption–desorption–regeneration cycles and results were given in Figure 6. After 10 adsorption–desorption–regeneration cycles, the p(HEMA-I) cryogel column showed no remarkable loss in hIgM adsorption and can protect its physical stability and high reusability ratios up to 97%. After the reusability studies, the p(HEMA-I) column was sterilized with a 50 mM NaOH solution and kept at 4 °C when used. 

## 3. Conclusions

Herein, we fabricated the p(HEMA) based immunoaffinity cryogel cartridge, which was synthesized under a semi-frozen condition for hIgM purification from aqueous solution and artificial human plasma with a single step. Total open porosity (percent) of the p(HEMA) cryogel column was found to be 95.2% and the macroporosity of p(HEMA) and p(HEMA-I) cryogel columns were examined with SEM.

The maximum adsorbed amount hIgM was calculated in the 11.10 mg/g polymer in a 5.75 MES buffer in aqueous solution and experimental results obeyed the Langmuir adsorption isotherm model with a good correlation R^2^ (0.99) value. Artificial human plasma was chosen as a natural source for hIgM purification and purity of hIgM in artificial human plasma was evaluated in SDS-PAGE. The same cryogel was used 10 adsorption–desorption–regeneration cycles without significant loss in adsorption capacity.

## 4. Materials and methods

### 4.1. Materials

Lyophilized hIgM, anti-hIgM, artificial plasma and ammonium persulfate (APS) were purchased from Sigma (St Louis, USA). N,N,N′,N′-tetramethylene diamine (TEMED), 2-hydroxyethyl methacrylate (HEMA), methylene bisacrylamide (MBAAM), hydroxyethylpiperazine ethane sulfonic acid (HEPES), MES and tris(hydroxymethyl)aminomethane hydrochloride (Tris-HCI) were obtained from Fluka A.G. (Buchs, Switzerland). Other chemicals used in experimental studies were all of reagent grade and were obtained from Merck AG (Darmstadt, Germany). In all experiments purified water was used by using a Barnstead (Dubuque, IA, USA) ROpure LP® reverse osmosis unit with a high-flow cellulose acetate membrane (Barnstead D2731) followed by a Barnstead D3804 NANOpure® organic/colloid removal and ion exchange packed-bed system. 

### 4.2. Preparation of p(HEMA-I) Cryogel Column

p(HEMA) cryogel columns were prepared as detailed following the process. HEMA (1.3 mL), as a monomer, and MBBAM (0.283 g) as a cross linker were dissolved in 15 mL of deionized water and then the polymer mixture was degassed to eliminate the oxygen for 5 min in the sonicator. Then 20 mg APS as an initiator was added in the polymer mixture, and then this solution was kept in an ice bath for 2-3 min. Finally, 25 µL of TEMED, as a stabilizator, was added in the solution. The solution was put in a plastic syringe and kept at −14 °C for 24 h. After polymerization, the cryogel column was thawed at room temperature and washed with deionized water to remove the impurities. After that, the p(HEMA) cryogel column was activated by using cyanamide [45]. Different amounts of anti-human hIgM (0.01–0.1 mg/mL) were immobilized onto the p(HEMA) based cryogel columns. The p(HEMA-I) cryogel column was washed with 200 mL distilled water and then stored in 0.02% (*w*/*v*) sodium azide solution at 4 °C until further use.

### 4.3. Preparation of Immunoaffinity p(HEMA-I) Cryogel Column

Cyanamide was used for the immobilization of anti-IgM [45]. Before the attachment of anti-IgM, the p(HEMA) cryogel column was sterilized with 0.1 M NaOH solution and then rinsed with deionized water. Briefly, the 5.0 mg/mL cyanamide solution was prepared (pH 7.4) then passed through the cryogel column for 24 h at room temperature at a flow rate (1 mL/min). Then the buffer solution (pH 7.4) was used to remove unreacted cyanamide.

### 4.4. Characterization Studies

#### 4.4.1. Properties of P(HEMA) and P(HEMA-I) Cryogel Columns Swollen in Water

Swelling degree (gH_2_O/g cryogel) and macroporosity (%) of cryogel columns were described in our previous studies [45,46]. The p(HEMA) and the p(HEMA-I) cryogel columns were dried at vacuum in an oven. Swelling degrees of the p(HEMA) and the p(HEMA-I) cryogel columns were determined by using Equation (1). The cryogel columns were washed with ethyl alcohol and distilled water until the washing solution was clear. Then the cryogel columns stayed in water until they were completely swollen and put on dried paper for the removal of excess water. Weights of the cryogel columns were determined. These cryogels were then dried at 55 °C and weighted (m_dry_; g).

Swelling degree (S) = (*m*_swollen_ – *m*_dry_) / *m*_dry_ (1)

Weights of the swollen cryogels (m_swollen_; g) were determined for the examination of macroporosity. Following removal of water from swollen cryogels by squeezing, the weights of the squeezed cryogels (m_squeezed_; g) were also determined. The macroporosity (%) of cryogels representing the volume fraction (in %) was determined by a method based on water vapor adsorption and was calculated according to Equation (2) [47,48].

(2) $$\mathrm{Macroporosity}\;\left(\%\right)=\frac{\left(m\mathrm{swollen}\;–\;mw\right)/\rho H2O\;}{\mathrm{mswollen}/\rho \mathrm{swollen}}\;\;\times \;100$$

where ρH2O is the density of water at 25 °C and ρ_swollen_ is the density of a swollen sample and wet samples swollen to their equilibrium in water vapor (m_w_).

#### 4.4.2. Characterization of the p(HEMA) and the p(HEMA-I) cryogel columns

Surface morphology, network structure of the p(HEMA) and the p(HEMA-I) cryogel columns were investigated with SEM. Before SEM analysis, the cryogel column was swelled in distilled water and kept at +4 °C for 24 h, then it was lyophilized at 0.0010 mbar, −55 °C for 12 h and kept at room temperature before the SEM analysis (Chris Alpha 1–2 LD Freeze Dryer, SciQuip, England). Afterwards, cryogels were coated with gold and their images were taken with different magnifications (JEOL, JEM, 1200 EX, Tokyo, Japan).

The p(HEMA) cryogel column was visualized by micro-computerized tomography (µ-CT Bruker, Skyscan 1272 USA). Before micro CT analysis, a lyophilized cryogel was dried at room temperature, then micro CT analysis was carried out and during the micro CT analysis, X-rays were sent to the samples at a 360° angle at a 0.4° interval on the cryogel’s surface and to enhance the image qualities, a 0.5 mm aluminum filter was used.

The p(HEMA) cryogel column was characterized by FTIR (Thermo Fisher Scientific, Nicolet iS10, Waltham, MA, USA). The samples were dried at 55 °C before analysis and the spectrum was taken between the 650 and 4000 cm^-1^ wave number.

### 4.5. Purification of hIgM from Aqueous Solution

hIgM binding experiments were performed in a recirculation system and before the adsorption process, the p(HEMA-I) cryogel column was equilibrated with an MES buffer because the maximum adsorption capacity was found in the MES buffer. After the equilibration of the MES buffer for 30 min, different amounts of hIgM molecules were passed through the p(HEMA-I) column for 2 h at room temperature. After the adsorption, a 1.0 M NaCI solution was passed through the p(HEMA-I) column to desorp the hIgM molecule on the cryogel column then the column was washed with distilled water.

The effect of pH (4.0–8.0), hIgM concentration (0.05–0.5 mg/mL), temperature (4–45 °C) and ionic strength (0–0.5 mM NaCl) parameters were examined and the adsorbed amount of hIgM was calculated with Equation (3) by using a UV-vis spectrophotometer at 280 nm (Shimadzu, 1601, Tokyo, Japan).

Q = (C_i_-C_f_). V/M (3)

Here, Q; describes the adsorbed amount of the hIgM molecule (mg/g), C_i_, C_f_; initial and final concentration of hIgM (mg/mL), V; volume of solution (mL) and M; weight of the adsorbent (g).

The Langmuir, the Freundlich and the Langmuir/Freundlich adsorption isotherm models were calculated with the experimental results [49]. The Langmuir, the Freundlich and the Langmuir/Freundlich binding isotherms can be defined as the Equation (4), (5) and (6), respectively.

Q = Q_max_b_L_C_e_/(1 + bC_e_) (4)

Q = K_F_ C_e_^1/n^ (5)

Q = Q_max_b_LF_C_e_^1/n^/(1 + b_LF_C_e_^1/n^) (6)

Here, Q is the adsorption capacity (mg/mL), C_e_ is the equilibrium hIgM concentration (mg/mL), b_L_ is the constant related to the affinity binding sites, K_F_ is the Freundlich constant and n is the Freundlich exponent. b_LF_ is the Langmuir –Freundlich isotherm constant. These parameters were calculated by applying non-linear regression methods to the experimental data.

### 4.6. hIgM Purification from Artificial Plasma

hIgM binding from artificial plasma with the p(HEMA-I) cryogel column was studied in a recirculation system. The artificial plasma was diluted to ½ and passed through the p(HEMA-I) cryogel column under recirculation for 2 h at 1 min/mL of the flow rate at room temperature. 

SDS-PAGE analysis of the plasma sample was carried out on 5% (*w*/*v*) stacking gel and 10% (*w*/*v*) separating mini vertical gel (9 cm × 7.5 cm; Mini-PROTEAN Tetra Cell, Bio-Rad) for 120 min at 100 V. This gel was stained with Coomassie Brilliant G 250 and destained in 10% *(v*/*v*) methanol solution. The gel was then visualized using an ImageQuant 300 (GE Healthcare, Buckinghamshire, UK) image analyzer. Prior to analysis, disulfide bonds of hIgM were broken by mercaptoethanol at 70 °C for 10 min. Analysis was then carried out [46] at room temperature.

### 4.7. Reusability of Cryogel Column

Reusability of the cryogel column is an important parameter in the purification process. Therefore, the reusability of the p(HEMA-I) cryogel column was evaluated with ten binding-elution cycles using the same cryogel column. The desorption agent was 1.0 M NaCl when the concentration of hIgM was 0.1 mg/mL. The p(HEMA-I) cryogel column was washed with 50 mM NaOH solution after each binding-elution cycle for regeneration and sterilization.

## Figures and Tables

**Table 1:** Physical properties of the p(HEMA) and the p(HEMA-I) cryogel columns.

Cryogel Samples	Swelling Degree (gH_2_O/g Cryogel)	Macroporosity (Volume %)
p(HEMA)	5.05	89.6
p(HEMA-I)	4.63	88.1

**Table 2:** The Langmuir, the Freundlich and the Langmuir–Freundlich binding isotherm constants.

Experimental	Langmuir Constants			Freundlich Constants			Langmuir–Freundlich Constants		
Q_max_ (mg/g)	Q_max_ (mg/g)	b_L_ (mL/mg)	R^2^	K_f_	*n*	R^2^	Q_max_ (mg/g)	K_LF_ (mL/mg)	R^2^	
11.10	11.83	45.37	0.99	11.24	75.18	0.89	10.1	1.1	0.97	

## Supplementary Materials

Supplementary materials can be found at https://www.mdpi.com/2310-2861/6/1/4/s1.
