# Supplementary material for: Poly(Hydroxyethyl Methacrylate) Immunoaffinity Cryogel Column for the Purification of Human Immunoglobulin M
Source: Gels. 2020 Jan 29;6(1):4. doi: 10.3390/gels6010004 (PMC7151037; doi:10.3390/gels6010004)
Supplement: Supplementary file 1 [file gels-06-00004-s001.pdf]

Supplementary

Poly(Hydroxyethyl Methacrylate) Immunoaffinity Cryogel Column for Purification of Human Immunoglobulin M

Monireh Bakhshpour [1], Aykut Arif Topcu [2], Nilay Bereli [1], Huseyin Alkan [3] and Adil Denizli [1,*]

[1] Department of Chemistry, Biochemistry Division, Hacettepe University, Ankara, Turkey
[2] Department of Chemistry, Aksaray University, Aksaray, Turkey
[3] Department of Chemistry, Dicle University, Diyarbakır, Turkey

* Correspondence: denizli@hacettepe.edu.tr; Tel.: +90 312 299 2163

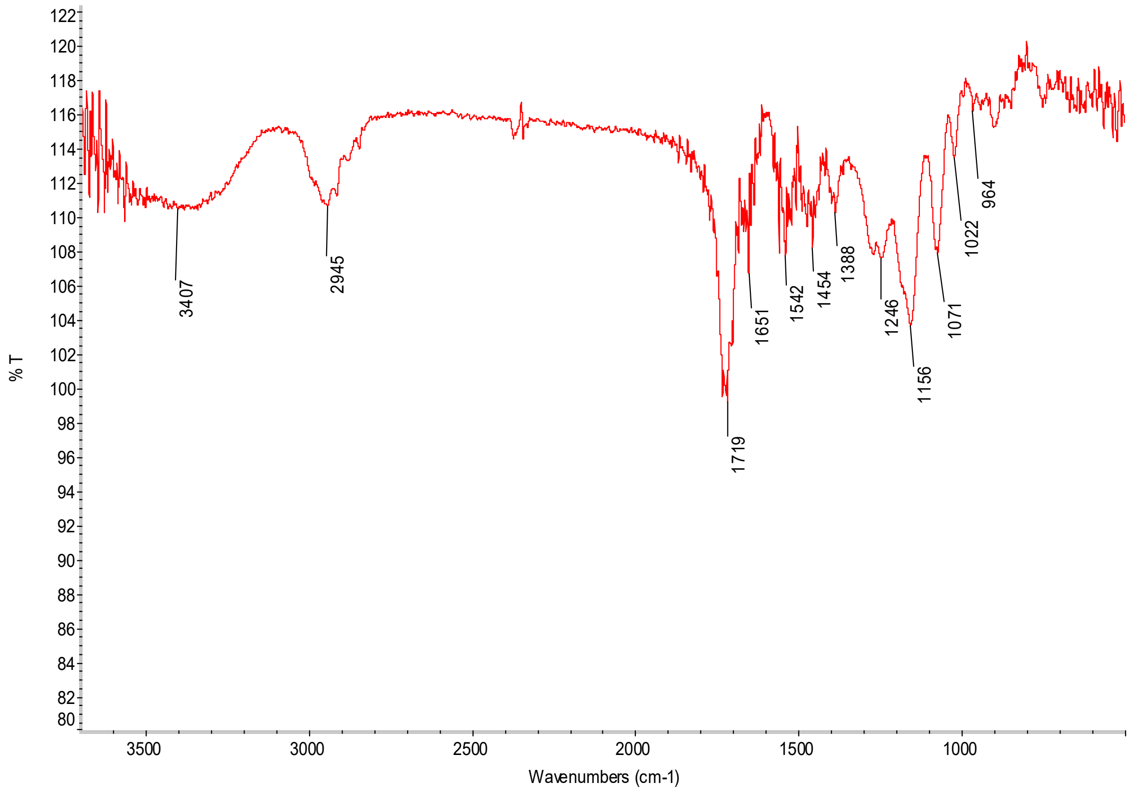

The FTIR spectrum of the p(HEMA) cryogel column, which have described as -OH stretching around 3407 cm^{-1}, aliphatic -CH strectching around 2945 cm^{-1}, carbonly C=O strectching around 1719 cm^{-1}, 1156 cm^{-1} (C-O strethching), the peaks around at 1651 cm^{-1} and 1542 cm^{-1} as described amide I (C=O streching), amide II (C=O streching), respectively, which were stemmed from cross-linker are the characteristic peaks of p(HEMA). The other peaks, ester bond around 1454 cm^{-1}, C-N bond from cross-linker around 1388 cm^{-1} and ether bonds arounds 1246 cm^{-1}, 1071 cm^{-1}, respectively. FTIR results of this work was in accordance with previous studies [42,43].

References

42. Attieh-Daoud, M; Chaib, H; Armutcu, C; Uzun, L; Elkak, A; Denizli, A; Immunoglobulin g purification from bovine serum with pseudo-specific macroporous cryogels. Sep. Purif. Technol. 2013, 118, 816-822.

43. Derazshamshir, A; Baydemir, G; Yılmaz, F; Bereli, N; Denizli, A. Preparation of cryogel columns for depletion of hemoglobin from human blood. Artif. Cell. Nanomed. B. 2016, 44, 3, 792-799.
